# A comparative study of four intensive care outcome prediction models in cardiac surgery patients

**DOI:** 10.1186/1749-8090-6-21

**Published:** 2011-03-01

**Authors:** Fabian Doerr, Akmal MA Badreldin, Matthias B Heldwein, Torsten Bossert, Markus Richter, Thomas Lehmann, Ole Bayer, Khosro Hekmat

**Affiliations:** 1 Department of Cardiothoracic Surgery, Friedrich-Schiller-University of Jena, Erlanger Allee 101, 07747 Jena, Germany; 2 Institute of Medical Statistics, Computer Sciences and Documentation, Friedrich-Schiller-University of Jena, Bachstrasse 18, 07743 Jena, Germany; 3 Department of Anesthesiology and Intensive Care Medicine, Friedrich-Schiller-University of Jena, Erlanger Allee 101, 07747 Jena, Germany

## Abstract

**Background:**

Outcome prediction scoring systems are increasingly used in intensive care medicine, but most were not developed for use in cardiac surgery patients. We compared the performance of four intensive care outcome prediction scoring systems (Acute Physiology and Chronic Health Evaluation II [APACHE II], Simplified Acute Physiology Score II [SAPS II], Sequential Organ Failure Assessment [SOFA], and Cardiac Surgery Score [CASUS]) in patients after open heart surgery.

**Methods:**

We prospectively included all consecutive adult patients who underwent open heart surgery and were admitted to the intensive care unit (ICU) between January 1^st ^2007 and December 31^st ^2008. Scores were calculated daily from ICU admission until discharge. The outcome measure was ICU mortality. The performance of the four scores was assessed by calibration and discrimination statistics. Derived variables (Mean- and Max- scores) were also evaluated.

**Results:**

During the study period, 2801 patients (29.6% female) were included. Mean age was 66.9 ± 10.7 years and the ICU mortality rate was 5.2%. Calibration tests for SOFA and CASUS were reliable throughout (p-value not < 0.05), but there were significant differences between predicted and observed outcome for SAPS II (days 1, 2, 3 and 5) and APACHE II (days 2 and 3). CASUS, and its mean- and maximum-derivatives, discriminated better between survivors and non-survivors than the other scores throughout the study (area under curve ≥ 0.90). In order of best discrimination, CASUS was followed by SOFA, then SAPS II, and finally APACHE II. SAPS II and APACHE II derivatives had discrimination results that were superior to those of the SOFA derivatives.

**Conclusions:**

CASUS and SOFA are reliable ICU mortality risk stratification models for cardiac surgery patients. SAPS II and APACHE II did not perform well in terms of calibration and discrimination statistics.

## Background

Scoring systems were introduced into intensive care medicine to provide the physician with an objective tool for judging a patient's condition and likely outcome. These scores can be used to estimate the severity of disease and to aid therapeutic decisions. The acute patho-physiological sequelae of cardiopulmonary bypass are transient and many physiologic changes may be masked by multiple system support devices, such as intra-aortic balloon pumps, ventricular assist devices, hemofiltration and mechanical ventilation. The subset of cardiac surgery patients was, therefore, excluded during the development of many general scoring systems, such as the Acute Physiology and Chronic Health Evaluation (APACHE) and the Simplified Acute Physiology Score (SAPS) [1,2]. Nevertheless, many of these scoring systems are used in cardiac surgery intensive care units (ICU) because of the lack of an appropriate risk index for this specific subgroup of patients. In central Europe, the most commonly used postoperative scoring systems in cardiac ICUs are APACHE II [1], SAPS II [2] and the Sequential Organ Failure Assessment (SOFA) [3]. Recently, the Cardiac Surgery Score (CASUS) [4] was introduced to specifically target cardiac surgery patients, but it is not yet widely used. In this study, we compared the mortality prediction of CASUS and the other well-known ICU scoring systems after cardiac surgery. The variables included in these four scores are shown in Table 1.

**Table 1:** Summary of variables included in the different postoperative scoring systems

Variables	CASUS	APACHE II	SAPS II	SOFA
**Cardiovascular system**				
Blood pressure	**√**	**√**	**√**	**√**
Heart rate	**√**	**√**	**√**	
CVP	**√**			
Lactate	**√**			
IABP	**√**			
VAD	**√**			
NYHA IV (cardiac)		**√**		
Catecholamines				**√**
**Respiratory system**				
Oxygenation	**√**	**√**	**√**	**√**
Respiratory rate		**√**		
COPD		**√**		
Hypoxia		**√**		
Hypercapnia		**√**		
Pulmonary hypertension		**√**		
Patient dependence on respirator		**√**		
**Renal system**				
Creatinine	**√**	**√**		**√**
Urine output			**√**	**√**
Dialysis	**√**	**√**		
Urea			**√**	
**Hepatic system**				
Bilirubin	**√**		**√**	**√**
Cirrhosis		**√**		
Portale hypertension		**√**		
GI bleeding		**√**		
Liver collapse		**√**		
Hepatic encephalopathy		**√**		
**Hematological system**				
Leukocytes		**√**	**√**	
Platelets	**√**			**√**
Hematocrit		**√**		
**Central nervous system**				
GCS		**√**	**√**	**√**
Neurologic state	**√**			
**Electrolyte/Metabolic status**				
Sodium		**√**	**√**	
Potassium		**√**	**√**	
Bicarbonate		**√**	**√**	
**Patient data**				
Age		**√**	**√**	
**Chronical disease**				
Metastasis/tumor		**√**	**√**	
Leukemia		**√**	**√**	
AIDS		**√**	**√**	
Therapeutic low immunity		**√**		
**ICU-admission**				
Elective surgery		**√**	**√**	
Internal disease		**√**	**√**	
Emergency OP		**√**	**√**	
**Others**				
Temperature		**√**	**√**	
pH		**√**		

CVP: central venous pressure; IABP: intra-aortic balloon pump; VAD: ventricular assist device; COPD: chronic obstructive pulmonary disease; GI: gastrointestinal; GCS: Glasgow coma scale; AIDS: acquired immunodeficiency disease.

## Methods

This study involved an evaluation of prospectively collected data from all consecutive adult patients admitted to our ICU after cardiac surgery. Patients admitted between January 1^st ^2007 and December 31^st ^2008 were included and the study was approved by the Institutional Review Board of Friedrich Schiller University Hospital (approval no.: 2809-05/10). Only the first admission was considered for patients who were readmitted to the ICU during the study period. Data were collected from the quality control system QUIMS 2.0b (University Hospital of Muenster, Germany) and from the intensive care information system COPRA 5.2 (COPRASYSTEM GmbH, Sasbachwalden, Germany), which is interfaced with patient monitors (Philips IntelliVue MP70, Amsterdam, Netherlands), ventilators (Draeger Evita IV, Luebeck, Germany and Hamilton Galileo, Bonaduz, Swizerland), blood gas analyzing devices (ABL 800Flex Radiometer, Copenhagen, Denmark) and the central laboratories.

The attending physician collected the study data of all scores for the first postoperative week. Two assigned medical clerks validated the data collection daily. A senior consultant performed a second periodical validation. Inconsistency between the raters was resolved by consensus. There were no missing data. Outcome was defined as ICU mortality. The scores were calculated using the most abnormal value for each variable per day. The maximum derivative of any scoring system (Max-score) was defined as the worst daily score throughout the whole ICU stay. Mean-score was calculated by dividing the sum of all daily values during the ICU stay by the ICU length of stay (ICULOS) in days.

### Statistical analyses

Statistical analyses were performed with SPSS software version 18 (SPSS Inc, Chicago, IL). Graphics were drawn using Microsoft Excel software. Continuous scale data are presented as mean ± standard deviation (SD) and were analyzed using the two-tailed Student's t-test for independent samples. The Kolmogorov-Smirnov test showed a normal distribution of the continuous data. A p value of < 0.05 was considered as significant. Calibration was performed using the Hosmer-Lemeshow (HL) test (goodness-of-fit-test) to insure the absence of a significant discrepancy between predicted and observed mortality. Calibration was considered good when there was a low χ2 value and a high p value (>0.05). Discrimination (ability of a scoring model to differentiate between survival and death) was evaluated with receiver-operating-characteristic (ROC) curves; the area under the curve (AUC) indicates the discriminative ability of the scores, i.e., the ability to discriminate survivors from non-survivors. AUCs enable direct comparison of different scoring systems: An AUC of 0.5 (a diagonal line) is equivalent to random chance, AUC >0.7 indicates a moderate prognostic model, and AUC >0.8 (a bulbous curve) indicates a good prognostic model. The overall correct classification (OCC) (the ratio of number of correctly predicted survivors and non-survivors to the total number of patients) values of the scores were calculated. The risk of mortality is given as odds ratios for all scores with 95%-confidence intervals. All statistical analyses were performed from ICU day 1 (n = 2801) (operative day) to day 6 (n = 431 patients) only, in order to obtain accurate statistical results and to avoid a small number of patients. The preoperative logistic and additive EuroSCORE were also statistically tested.

## Results

The study included 2801 patients who were admitted to the ICU over the two-year period; 29.6% (n = 830) were female, and mean age was 66.9 ± 10.7 years (range of 19-89 years). The types of surgical procedures are shown in Table 2. ICULOS was 4.3 ± 6.8 days (range 1-189 days, median 2.0 days, 75^th ^percentile 4.0 days) and ICU mortality was 5.2% (n = 147). The preoperative collected mean additive EuroSCORE was 6.3 ± 3.6 and the mean logistic EuroSCORE was 9.9 ± 12.9 (median 5.3, 75^th ^percentile 11.3).

**Table 2:** Type of surgery in the study population

Operation	number	%
CABG	1526	54.5

Isolated valve surgery	635	22.7

Combined CABG & valve surgery	381	13.6

Ascending aorta and aortic arch surgery	60	2.1

Combined ascending aorta & valve surgery	116	4.1

Combined ascending aorta & coronary surgery	5	0.2

Cardiac transplantation	24	0.9

Congenital, cardiac tumors, pulmonary embolectomy, Assist device implantation	54	1.9

**Total**	**2801**	**100**

CABG: Coronary artery bypass grafting.

**Figure 1:**
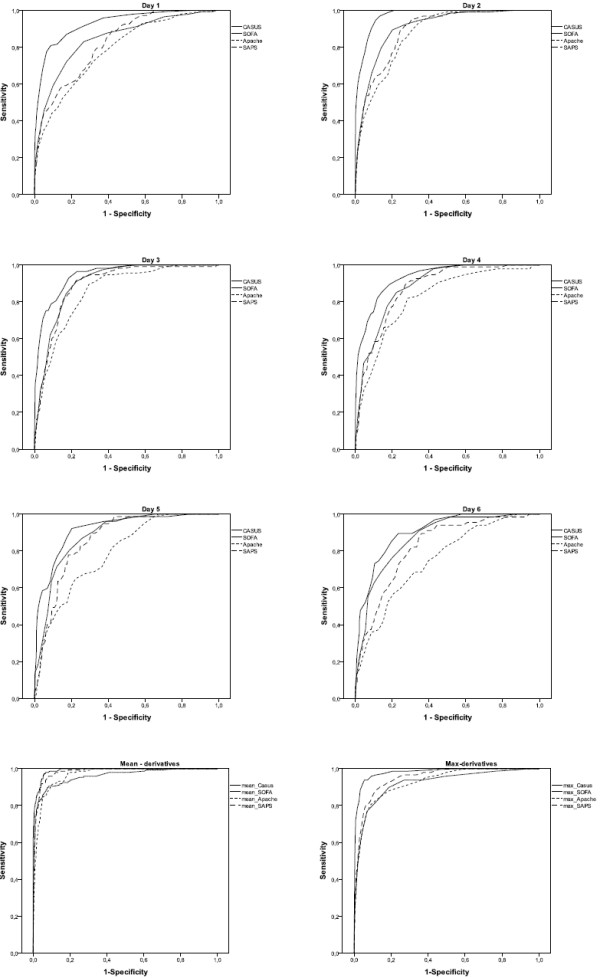
**Day 1-6: ROC-curves of CASUS, SOFA, APACHE II, SAPS II and their derivatives**.

**Figure 2:**
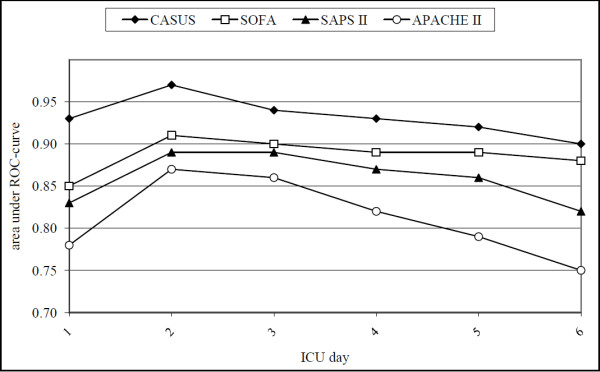
**Day 1-6: Areas under the ROC-curves of CASUS, SOFA, APACHE II and SAPS II**.

Table 3 summarizes the OCC, calibration and discrimination of all four models from the first ICU day to day 6 and for both preoperative EuroSCORE models. There were no significant differences between expected and observed mortality for CASUS, SOFA and the preoperative additive EuroSCORE using the HL-test, but there were differences for the preoperative logistic EuroSCORE (p = 0.01), SAPS II (p < 0.05 on ICU admission and days 2, 3 and 5) and APACHE II (p < 0.05 on days 2 and 3). Figure 1 shows the ROCs of all the postoperative models for the first six ICU days. The AUC for CASUS (≥ 0.90) was greater than those of the other scoring systems on all studied days; the largest AUC was achieved with CASUS on the second ICU day (AUC = 0.97) (Table 3, Figure 2). SOFA performed better than APACHE II and SAPS II in this statistical analysis. The OCC was greater for CASUS than for the other scores on all days with the best result on the second ICU day (OCC = 96.9%).

**Table 3:** Day 1-6: Logistic regression, OCC, calibration (HL), discrimination (ROC) for EuroSCORE, CASUS, SOFA, SAPSII, APACHEII

Day	Scoring model	Logistic Regression		OCC	HL test		ROC-Analysis	
		
		O R	95%-CI	%	χ^1^	p-value	AUC	95%-CI
**Preoperative (2801)**	**Add-Euro**	1.25	1.20-1.30	94.7	9.10	0.33	0.71	0.64-0.79
	
	**Log-Euro**	1.04	1.03-1.05	94.7	**19.75**	**0.01**	0.71	0.63-0.78

	**CASUS**	1.55	1.48-1.64	96.0	3.65	0.82	0.93	0.91-0.95
	
**ICU-Day 1 (2801)**	**SOFA**	1.70	1.58-1.82	95.3	7.90	0.34	0.85	0.81-0.88
	
	**SAPS II**	1.08	1.07-1.10	95.0	**36.60**	**<0.001**	0.83	0.79-0.86
	
	**APACHE II**	1.17	1.14-1.19	95.0	5.28	0.626	0.78	0.75-0.82

	**CASUS**	1.50	1.43-1.58	96.9	13.97	0.05	0.97	0.96-0.98
	
**ICU-Day 2 (2769)**	**SOFA**	1.64	1.54-1.76	95.3	6.75	0.56	0.91	0.88-0.93
	
	**SAPS II**	1.09	1.08-1.10	95.4	**33.87**	**<0.001**	0.89	0.87-0.91
	
	**APACHE II**	1.20	1.17-1.23	95.3	**30.63**	**<0.001**	0.87	0.85-0.90

	**CASUS**	1.37	1.31-1.43	93.8	10.29	0.17	0.94	0.93-0.96
	
**ICU-Day 3 (1234)**	**SOFA**	1.55	1.44-1.66	90.8	6.45	0.60	0.90	0.88-0.93
	
	**SAPS II**	1.09	1.08-1.10	90.9	**17.15**	**0.03**	0.89	0.87-0.92
	
	**APACHE II**	1.20	1.16-1.23	91.0	**18.13**	**0.02**	0.86	0.83-0.89

	**CASUS**	1.36	1.29-1.43	92.4	3.66	0.82	0.93	0.91-0.96
	
**ICU-Day 4 (815)**	**SOFA**	1.50	1.39-1.62	89.3	8.35	0.40	0.89	0.86-0.91
	
	**SAPS II**	1.08	1.07-1.10	89.3	12.18	0.143	0.87	0.84-0.91
	
	**APACHE II**	1.18	1.14-1.22	88.6	8.42	0.297	0.82	0.78-0.86

	**CASUS**	1.34	1.26-1.41	91.2	8.08	0.33	0.92	0.89-0.95
	
**ICU-Day 5 (566)**	**SOFA**	1.51	1.39-1.65	86.9	2.46	0.96	0.89	0.85-0.92
	
	**SAPS II**	1.08	1.06-1.09	86.0	**18.99**	**0.015**	0.86	0.83-0.90
	
	**APACHE II**	1.16	1.12-1.20	86.2	14.30	0.07	0.79	0.74-0.84

	**CASUS**	1.32	1.25-1.41	89.5	4.71	0.79	0.90	0.86-0.94
	
**ICU-Day 6 (430)**	**SOFA**	1.47	1.35-1.61	85.6	3.98	0.86	0.88	0.84-0.91
	
	**SAPS II**	1.07	1.05-1.08	85.6	5.11	0.75	0.82	0.77-0.87
	
	**APACHE II**	1.14	1.10-1.19	85.8	5.96	0.65	0.75	0.69-0.81

95%-CI: 95%-confidence interval, Add-Euro: additive EuroSCORE, AUC: Area under ROC curve, HL: Hosmer-Lemeshow, Log-Euro: logistic EuroSCORE, OCC: overall correct classification, CC: tio for risk of mortality, OR: Odds ratio for risk of mortality, ROC: receiver operating characteristic.

Table 4 shows the results for the statistical evaluation of the score-derivatives. There were no significant differences between expected and observed mortality using the HL-test. CASUS again had the best discrimination. In the ROC test, in contrast to the results for the original scores, the derivatives of SAPS II and of APACHE II performed better than the derivatives of SOFA. All derived scores had higher OCCs than the original scores.

**Table 4:** Logistic regression/odds ratio, OCC, calibration (HL), discrimination (ROC) for CASUS, SOFA, SAPSII, APACHEII derivatives

Derivative of the Scoring model	Logistic Regression		OCC	HL test		ROC-Analysis	
	
	O R	95%-CI	%	χ^1^	p-value	AUC	95%-CI
**Mean-CASUS**	2.04	1.85-2.62	98.3	5.75	0.68	0.991	0.987-0.995

**Mean-SOFA**	2.76	2.43-3.12	97.3	11.33	0.18	0.96	0.94-0.98

**Mean-SAPS II**	1.26	1.22-1.29	97.2	5.22	0.73	0.982	0.975-0.988

**Mean-APACHE II**	1.64	1.54-1.75	96.2	3.78	0.88	0.97	0.96-0.97

**Max-CASUS**	1.60	1.51-1.70	97.8	2.12	0.95	0.98	0.97-0.99

**Max-SOFA**	1.81	1.69-1.95	95.6	10.54	0.16	0.92	0.90-0.95

**Max-SAPS II**	1.15	1.13-1.18	95.9	3.75	0.88	0.95	0.94-0.96

**Max-APACHE II**	1.35	1.30-1.40	95.5	14.38	0.07	0.93	0.91-0.95

95%-CI: 95%-confidence interval, AUC: Area under ROC curve, HL: Hosmer-Lemeshow, OCC: overall correct classification, OR: Odds ratio for risk of mortality, ROC: receiver operating characteristic.

## Discussion

Patients undergoing cardiac surgery show temporary pathophysiological effects related to the heart-lung-machine [5,6] that can influence the values of the postoperative scoring systems [7] and may make them unreliable in this population. These effects include the relatively long mechanical ventilation time needed to stabilize these patients [8,9] and the postoperative sedation that limits the role of the Glasgow Coma Scale (GCS) as a prognostic parameter [10]. Electrolyte- and blood glucose imbalances are also frequent [4]. All these factors are temporary and have a limited effect on prognosis. In addition, most currently used scoring systems ignore some of the parameters that can influence outcomes in these patients. The most common examples of this are the use of intra-aortic balloon pumps (IABP) and ventricular assist devices (VAD), and the presence of postoperative low cardiac output syndrome (LCOS) [5,6,8,11]. In 2005, CASUS [4] was suggested as a specialized cardiac surgery scoring system that took into account the special circumstances encountered in the ICU after cardiac surgery. However, many ICUs are still using the general postoperative risk stratification models for cardiac surgery patients, notably, in central Europe, the SOFA, APACHE II and SAPS II scores. Postoperative risk stratification is increasingly used, especially in cardiac surgery, and we believed it was important to compare these widely used scoring systems with the relatively new model (CASUS) to try and identify the optimal tool in this field.

The APACHE II model [1], published in 1985, was developed to simplify the original APACHE model and has become the most frequently used general mortality prediction model. APACHE II has been extensively validated, and despite being the oldest system, it still performs well [12]. More recent versions (APACHE III and IV) have not been widely adopted. All the APACHE models are based on the most abnormal values registered during the first 24 h after ICU admission. However, because several studies [13,14] have supported serial daily usage of postoperative risk stratification models, we chose to evaluate APACHE II on all ICU days. In our study, APACHE II had the worst discrimination of the four models studied but its calibration was better than that of SAPS II.

SAPS II was developed in 1994 [2] based on a European/North American database, which included 13,152 patients. Logistic regression analysis was used to select variables, and for weighting and conversion of the score to give the probability of hospital mortality for ICU patients over the age of 18. Although cardiac surgery patients were originally excluded from the score's target, it is used in many cardiac ICUs. SAPS II has been extensively studied and validated. There seems to be quite convincing evidence of the ability to maintain good discrimination across different populations, but calibration is often poor [15,16]. Our study in cardiac surgery patients, confirmed the poor calibration of SAPS II and its discrimination was worse than that of SOFA and CASUS. SAPS III [17] was introduced in 2005 in an attempt to overcome shortcomings related to different case-mixes and lead-time bias of SAPS II. However, its calibration and discrimination set were shown to vary widely around the world [12] so that many centers in central Europe still use the older version.

The SOFA was originally developed in 1996 as a morbidity risk stratification model for patients with sepsis [3]. Because of its good performance and reliability, SOFA is widely used as a scoring model for ICU patients not only for morbidity but also for mortality prediction [7]. In 2003, Ceriani et al. [14] suggested the use of SOFA in cardiac surgery patients. Based on the good results they obtained in 218 patients, they concluded that SOFA was applicable in cardiac surgery without any need for specific modifications. SOFA comprises separate daily scores for respiratory, renal, cardiovascular, central nervous, coagulation, and hepatic systems. The scores can be used in several ways, as individual scores (for each organ), as the sum of scores on a single ICU day, or as the sum of the worst scores during the ICU stay.

CASUS was developed based on retrospective analyses to identify descriptors of mortality and multiorgan dysfunction in postoperative cardiac surgical patients. It was then evaluated prospectively in 3230 patients in a single center study [4]. The main goal was to develop a scoring model that was specific to this type of patient and had a minimum number of descriptors. CASUS is, therefore, a compact score index with only ten, readily available descriptors. This scoring system has not yet been externally validated in multicenter studies, and accordingly, has not yet gained much popularity.

The ideal scoring system should not only be simple and reproducible but also reliable. This reliability can be assessed using calibration and discrimination tests, considered by the European Society of Intensive Care Medicine (ESICM) to be the best methods to validate score systems and prognostic parameters [18]. It has been argued that perfect discrimination is important in order to evaluate an individual patient's risk using a scoring system, whereas for clinical trials or comparison of care between ICUs better calibration is needed [19]. Accordingly, validations of scoring systems in the literature have frequently been achieved using good discrimination tests, although the HL test has often resulted in unreliable calibration. The HL test is very sensitive to the size of the study population with large numbers of patients resulting in unreliable calibration [20]. This fact is applicable to study populations larger than 5000 patients [20], which was not the case in our study. In other words, if the HL-test, in studies with more than 5000 patients, is significant this does not necessarily mean that the scoring systems are not useful or are unreliable [20].

However, our study, with a more optimal size of study population, showed that APACHE II and SAPS II are not suitable for use in cardiac surgery patients. CASUS and SOFA had an acceptable performance with the HL-test compared to the other two scores. CASUS was clearly superior in its ability to discriminate between survival and death on all days. This predictive property allows complications to be anticipated in individual patients and should alert residents, especially those with relatively little experience, to ask for help. The OCC (the ratio of correctly predicted number of survivors and non-survivors to the total number of patients) was also better in CASUS than in the other scores. We decided not to compare the different scores using odds ratios, because conclusions from such analyses can be distorted, as the maximum points in the different scoring systems vary significantly. Nevertheless odds ratios are useful tools to estimate the risk of mortality. Hence, for example, results can be influenced by different inotropic regimes or fluid replacement strategies in different hospitals. The assessment of the central nervous system may also affect results because the GCS is affected by sedation, anesthesia and paralysis [10,21,22], and calculation requires clinical evaluation, which may be biased by subjective interpretation [6,10,22]. CASUS is not affected by these problems. Its simple variable, 'neurologic state', can be calculated in less than one minute per patient per day. The parameters included in any scoring system influence its usefulness in different populations of patients. It is, therefore, perhaps not surprising that CASUS, which was specifically constructed for cardiac surgery patients, is superior to general severity systems in this group.

Mean- and max-score derivatives were introduced for SOFA by Moreno et al. [23] in 1999 and Ferreira et al. [24] in 2001. These methods were extended by Ceriani et al. [14] in 2003. We chose to calculate mean- and max-values for all four scores. However, it should be remembered that calculating the mean- and max-values adds some degree of selectivity to the model. The mean-derivative of a model reflects the overall average, whereas the max-derivative highlights the peak of organ dysfunction during the postoperative ICU stay; both are associated with the ICULOS, and thus allow a defined outcome prediction. The mean- and max-derivatives of all scores demonstrated better calibration, discrimination and OCC than the original models.

Similar to other studies, we detected a severe decrease in the study population on the third day because uncomplicated cases had been transferred to the general floor (Table 3). It is therefore important that a score is reliable during the first two days so that patients at risk are not discharged too early potentially leading to ICU-readmission and/or prolonged hospital stay, both of which are associated with higher mortality rates [25,26]. The good prognostic abilities of SOFA and CASUS in this study suggest they could be used to identify high-risk patients, enabling certain precautions to be put into place, such as daily monitoring of physiological dysfunction [27], and allowing prognoses and therapeutic choices, including withdrawal of therapy, to be discussed and reconsidered [28]. Nevertheless, no scoring system can replace clinical evaluation at a patient's bedside; they can only serve as an objective tool in decision making. Although scoring systems may provide an indication of disease severity and prognosis in individual patients and assist in overall patient assessment along with full clinical evaluation and other available parameters, they are designed for use in groups of patients and should never be the sole basis for therapeutic decisions [29].

## Conclusion

SOFA and CASUS are reliable tools for risk stratification in cardiac surgery patients. CASUS is more accurate than SOFA in mortality prediction. In contrast, APACHE II and SAPS II are not the tools of choice for this group of patients.

## Competing interests

The authors declare that they have neither a financial nor a non-financial competing interest.

## Authors' contributions

FD: substantial contributions to conception and design; acquisition, analysis and interpretation of data; drafting the manuscript. AB: substantial contributions to conception and design; revising the manuscript critically for important intellectual content. MH: acquisition and analysis of data; revising the manuscript it critically for important intellectual content. TB: final approval of the version to be published. MR: revising the manuscript critically for important intellectual content; final approval of the version to be published. TL: substantial contributions to statistical methods and analyses. OB: final approval of the version to be published. KH: substantial contributions to conception and design; interpretation of data; revising the manuscript critically for important intellectual content. All authors read and approved the final manuscript.
